# Capacitance-Driven Modulation of Cardiac Impulse Conduction by an Intramembrane Molecular Photoswitch

**DOI:** 10.3390/ijms262411766

**Published:** 2025-12-05

**Authors:** Chiara Florindi, Alessio Ostini, Chiara Bertarelli, Jan P. Kucera, Francesco Lodola

**Affiliations:** 1 Department of Biotechnology and Biosciences, University of Milano-Bicocca, 20126 Milan, Italy; c.florindi@campus.unimib.it; 2 Center for Nano Science and Technology, Istituto Italiano di Tecnologia, 20134 Milan, Italy; chiara.bertarelli@polimi.it; 3 Department of Physiology, University of Bern, 3012 Bern, Switzerland; alessio.ostini@unibe.ch (A.O.); jan.kucera@unibe.ch (J.P.K.); 4 Graduate School for Cellular and Biomedical Sciences, University of Bern, 3012 Bern, Switzerland; 5 Department of Chemistry, Materials and Chemical Engineering “Giulio Natta”, Politecnico di Milano, 20133 Milan, Italy

**Keywords:** capacitance modulation, azobenzene photoswitch, cardiac impulse propagation

## Abstract

Membrane-targeted photoswitches are emerging as innovative tools to modulate cardiac excitability with high spatiotemporal precision. Ziapin2, a membrane-integrating azobenzene derivative, undergoes light-driven trans–cis isomerization that alters membrane capacitance (C_m_). In its trans configuration, Ziapin2 increases C_m_, while illumination relaxes the membrane and restores C_m_ toward baseline. Here, we investigated whether Ziapin2 can modulate conduction velocity (CV) in strands of neonatal or fetal murine cardiomyocytes cultured on microelectrode arrays. In the dark, trans-Ziapin2 significantly reduced CV, consistent with increased capacitive load slowing action potential propagation. Unexpectedly, photostimulation further decreased CV, likely reflecting the documented transient, capacitive-driven perturbations of the membrane potential, occurring without alterations in cellular conductances. These findings suggest that non-genetic light modulation of membrane capacitance can influence cardiac conduction and establish Ziapin2 as a novel optical tool to modulate cardiac impulse propagation.

## 1. Introduction

Conduction velocity (CV) is a fundamental parameter in cardiac electrophysiology, as it critically determines impulse propagation and arrhythmia dynamics [1,2]. Current approaches to modulate CV rely predominantly on pharmacological interventions (e.g., sodium channel blockers or gap junction modulators) [2,3,4,5,6], genetic manipulation (e.g., ion channel overexpression or silencing, optogenetic actuators) [7,8,9,10,11,12,13,14,15], or thermal strategies (e.g., localized heating or cooling) [16,17,18] each of which suffers from limited temporal and spatial precision, reversibility, or translational potential.

**Figure 1:**
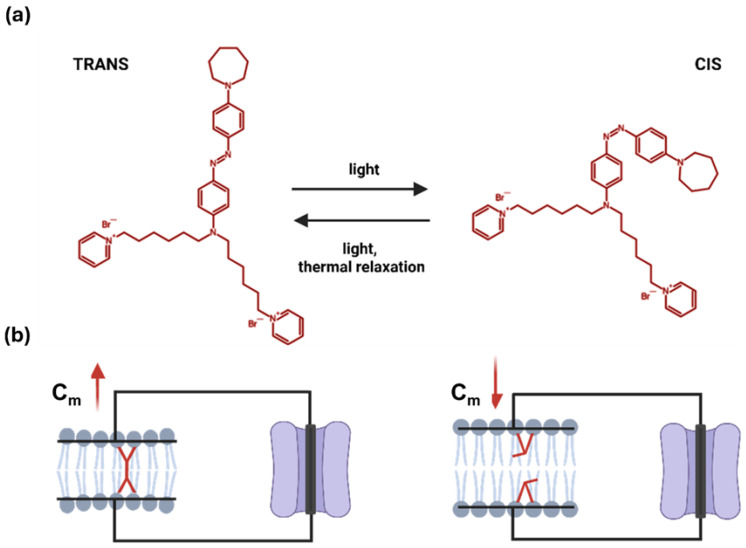
Schematic representation of the light-driven trans–to–cis isomerization of Ziapin2. (**a**) Molecular structure of Ziapin2 and its light-triggered shift between conformations. (**b**) In the trans configuration, Ziapin2 molecules span opposite sides of the membrane and form dimers, which locally thin the membrane and thereby increase its capacitance (left). When millisecond pulses of visible light (λ = 470 nm) are applied, Ziapin2 converts to the cis form. In this state, the hydrophobic chains of opposing molecules are separated too far to dimerize, leading to membrane relaxation and a rapid decrease in capacitance (right). Created in BioRender. Florindi, C. (2025) https://BioRender.com/0tq6ypk (accessed on 29 November 2025).

Light-based approaches independent of optogenetics overcome some of these limitations, offering further potential to influence cellular bioelectricity [19,20,21,22]. Among these, membrane-targeted photoswitches non-covalently bound to the plasma membrane are particularly attractive, as they can modulate membrane biophysical properties without requiring genetic modification or covalent tethering to specific proteins [23,24,25]. In this context, Ziapin2, a newly synthesized membrane-integrating azobenzene derivative, has emerged as a promising photochrome [26]. Ziapin2 partitions into the lipid bilayer and undergoes light-driven trans–cis isomerization. In its trans configuration, the molecules facing from the two phospholipid leaflets dimerize, thinning the membrane locally and increasing cellular capacitance (C_m_). Upon illumination with visible light (470 nm), dimers dissociate, leading to membrane relaxation and a rapid recovery of C_m_ toward baseline values (Figure 1).

Building on cable theory, even modest changes in C_m_ are predicted to affect conduction at the multicellular level, since an increased capacitive load alters the source-to-load relationship and slows impulse spread [1]. Leveraging this concept, we investigated whether Ziapin2 can modulate cardiac conduction through its action on membrane capacitance. To this aim, we employed strands of neonatal or fetal murine cardiomyocytes cultured on microelectrode arrays (MEAs), a well-established in vitro model that preserves key features of tissue-level conduction, including reproducible CV values and homogeneous propagation dynamics [27,28,29].

Our results reveal that Ziapin2 significantly reduces CV already in the dark, consistent with its effect in the trans state. Upon photostimulation, an additional paradoxical slowing was observed, most likely reflecting the transient rapid modulation of the membrane potential previously characterized as a capacitive response, independent of canonical ionic currents [26]. Overall, these findings support the notion that non-genetic modulation of membrane capacitance can influence conduction in cardiac tissue and establish membrane-targeted photoswitches as a novel candidate class of optical tools for tuning excitability without altering ionic conductances.

## 2. Results

Neonatal or fetal wild-type murine cardiomyocytes were patterned into aligned strands on microelectrode arrays (MEAs). After 2–3 days in culture, the preparations were incubated with Ziapin2 (25 µM) for 7 min in the dark (at 36 °C and 0.9% CO_2_), followed by washout and temperature equilibration for at least 1 h under the same incubating conditions.

**Figure 2:**
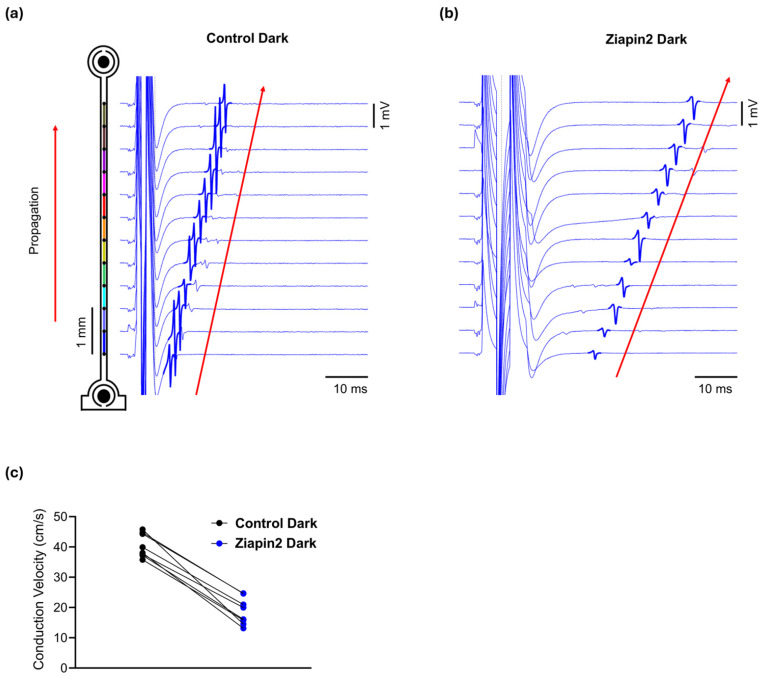
Ziapin2 reduces conduction velocity (CV) in cardiac strands in the dark. Representative extracellular field potential traces from MEA electrodes recorded before (**a**) and after Ziapin2-treatment (**b**) in a cultured strand electrically paced in the dark. Traces are vertically aligned according to electrode position along the series of electrodes (schematic on the left). In the presence of Ziapin2, a slower activation sequence was observed, with increased latency and reduced slope of field potential deflections consistent with decreased CV from 44.1 to 25.2 cm/s. (**c**) CV under dark conditions in Control and Ziapin2-treated strands. Data represents the CV measured in cardiac strands before light stimulation, showing a reduction in conduction speed upon Ziapin2 treatment compared to control.

We first assessed the effect of Ziapin2 on CV in the absence of light. Electrical stimulation was delivered via the MEA electrodes to pace the cardiac strands at a controlled cycle length of 300 ms (i.e., 3.33 Hz) during the recordings (see methods). In the dark, when Ziapin2 is in its trans configuration, we observed a significant reduction in CV compared to paired control recordings obtained before the application of Ziapin2 (Control: 40.41 ± 1.39 cm/s; Ziapin2-dark: 18.74 ± 1.58 cm/s; *n* = 8; *p* < 0.0001), with an average decrease of ~46% (Figure 2). This result is consistent with prior studies showing that trans-Ziapin2 significantly increases C_m_ in cardiomyocytes [30,31], thereby shifting the source-sink relationship towards the capacitive load and impairing conduction. Notably, in the presence of Ziapin2, higher electrical stimulation intensities and pulse durations were required to evoke propagated action potentials in comparison to control conditions, in agreement with the increased capacitance and the resulting decrease in excitability.

**Figure 3:**
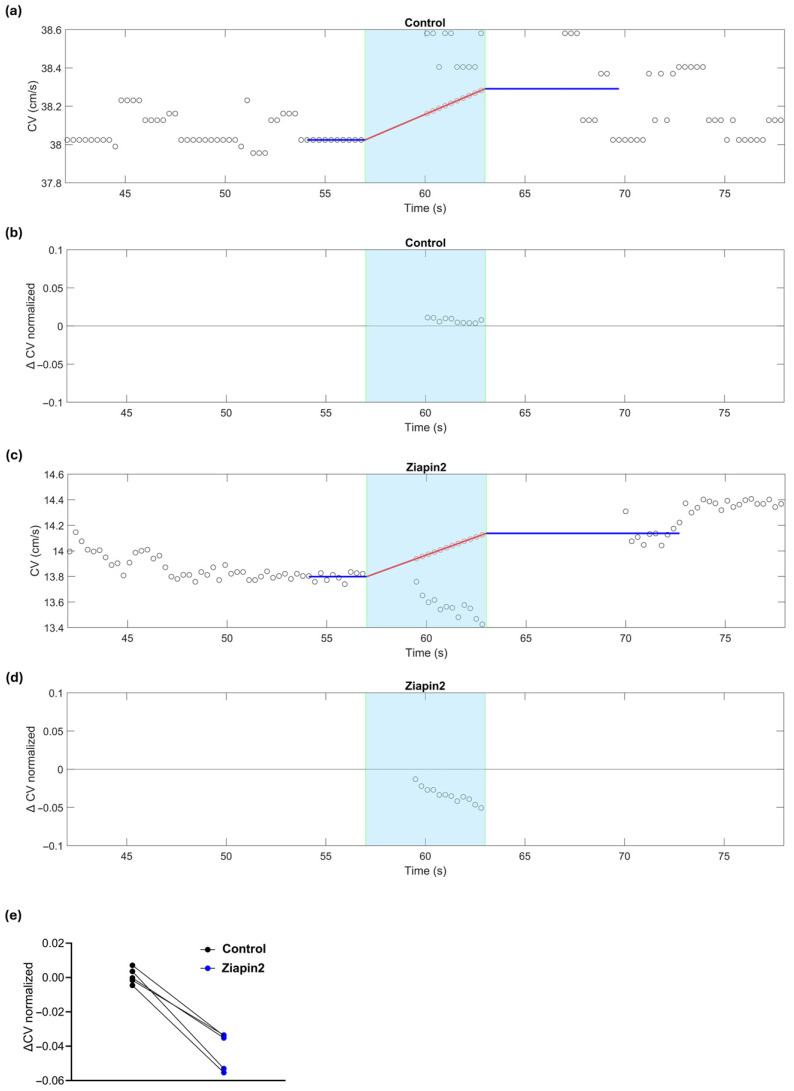
Effects of continuous light stimulation on CV in a cardiac strand before (control) and after Ziapin2-treatment. The cyan background marks the light exposure window (continuous light, 6 s). (**a**) CV as a function of time before Ziapin2 application. The blue lines represent the average/extrapolation of CV before and after illumination, respectively. The red line represents the linear interpolation of the CV trend during illumination (thermal effects). This extrapolation and interpolation of CV was necessary because turning the illumination on and off caused a transient (3–4 s) drift of the field potential that resulted in saturation of the amplifiers, which precluded CV measurement. (**b**) CV variation relative to the thermal prediction in the same strand, showing no significant deviation during illumination from the predicted interpolation. (**c**) Same as in panel (**a**) in the same strand after Ziapin2 treatment. Despite the thermal prediction indicating an increase in CV, a transient decrease was observed during light exposure relative to the interpolation line. (**d**) CV variation in the same Ziapin2-treated strand, showing a clear negative deflection from the thermal prediction during illumination, consistent with a photo-induced decrease of CV. (**e**) Data represents the ΔCV (relative to the interpolation of the thermal prediction) measured in cardiac strands subjected to continuous (6 s) light stimulation.

We then investigated the effects of continuous and pulsed illumination in Ziapin2-treated strands. Optical stimulation was delivered using a high-power 470 nm LED mounted inside the incubator and positioned beneath the transparent MEA, providing upward illumination of the cardiac strands. The light beam covered a circular area of approximately 1 cm diameter (78.5 mm^2^) on the MEA surface, allowing us to reach a maximum power density of ~12 mW/mm^2^ (Figure S1). At this intensity, in the absence of electrical stimulation, illumination did not elicit any extracellular electrograms indicative of action potentials in 5 preparations (Figure S2). To assess the direct impact of light itself without Ziapin2, we first evaluated control strands, since thermal heating from the LED alone could potentially affect CV. In these control strands, a small increase in CV was observed upon continuous light stimulation (from 39.8 ± 1.35 cm/s to 40 ± 1.39 cm/s; *n* = 9; *p* = 0.9494), consistent with an increase in temperature, which we measured to amount to approximately 0.3 °C per second in the culture bath (Figure 3a). This increase in CV aligned with the prediction obtained by extrapolating the CV trend before and after illumination and estimating the expected rise during illumination due to sample heating at a constant rate (red line in Figure 3a), suggesting that heat was the primary factor driving the change in CV. This discrepancy is highlighted in Figure 3b, where CV variation from the thermal prediction is plotted and no significant deviation is seen during illumination.

In contrast, Ziapin2-treated cultures subjected to 6 s illumination showed a modest but reproducible decrease in CV of ~4%, significantly diverging from thermal predictions (Figure 3c). While a temperature increase is expected to accelerate conduction (positive slope of the red line in Figure 3c), the experimental data fall clearly below this trend (Figure 3d), confirming a photo-induced decrease of CV consistently observed in all analyzed strands (*n* = 5, *p* = 0.0079, Figure 3e).

To further dissect the effects of Ziapin2 on conduction dynamics, we analyzed changes in conduction time between adjacent electrodes (spacing: 0.5 mm) in response to pulsed illumination (5–20 ms pulses, applied in the temporal window during which the action potential wavefront was propagating over the series of electrodes), comparing the situation before (control) and after Ziapin2 application. These very short light pulses are permitted to mitigate the development of heat. Moreover, the short duration of the light pulses also prevented the saturation of the amplifiers. These light pulses were applied 20 times, once every fifth electrical stimulus (i.e., once every 1.5 s at the pacing cycle length of 300 ms). This protocol permitted us to directly compare conduction times at every interelectrode interval in the presence vs. absence of light. For this purpose, the relative conduction time variation was calculated as the difference between the conduction time in the presence of a light pulse and the average conduction time of the 4 preceding and 4 following propagating action potentials, and this difference was normalized by this average. Hence, a value of 0.1 indicates an increase in conduction time by 10% (i.e., a decrease of CV by ~9.1%).

**Figure 4:**
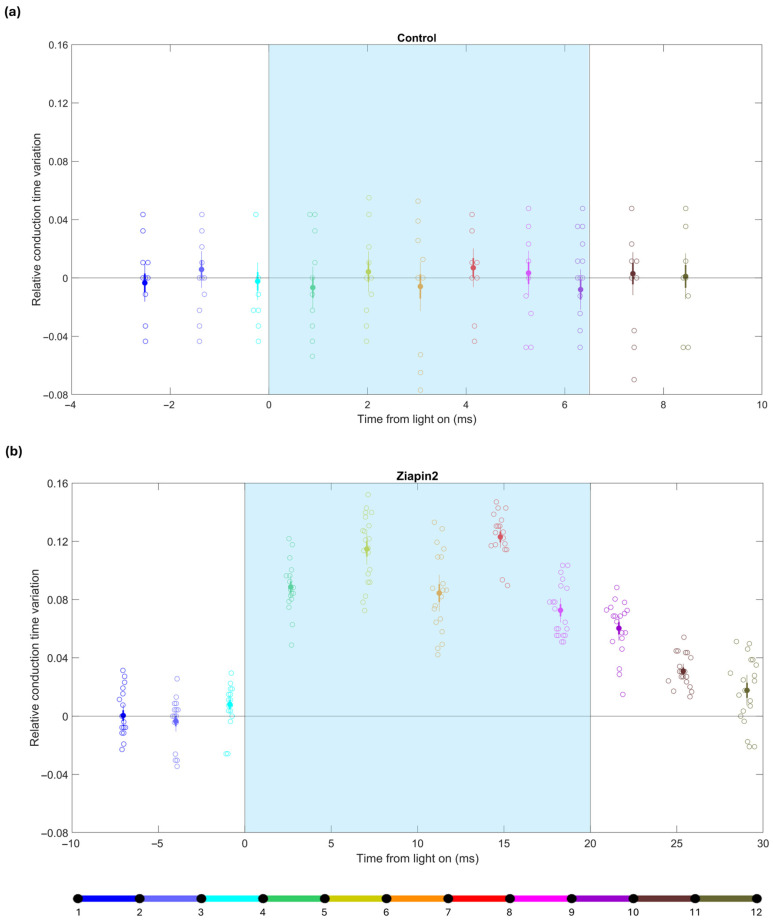
Relative conduction time variations during pulsed light stimulation in a cardiac strand before (control) and after treatment with Ziapin2. (**a**) Relative conduction time variations in the control experiment with pulsed light stimulation. Each data point represents a single illumination event. The x-axis represents the time at which the wavefront passed the center of the corresponding interval (relative to the onset of light), and the data points are color-coded according to the schematic of the 12-electrode and 11-interval layout shown at the bottom (see also Figure 2). Solid dots indicate means; thick error bars indicate ±1 S.E.M.; thin error bars indicate ±2 S.E.M. (*n* = 20 illumination pulses). (**b**) Relative conduction time variation in the same strand after treatment with Ziapin2 under similar pulsed light conditions. Color coding as in panel (**a**). The cyan shaded area indicates the light stimulation window. In this particular strand, the light was turned on when the wavefront was in the interval between electrodes 4 and 5 (green) and was turned off when the wavefront was between electrodes 8 and 9 (magenta).

In control conditions (Figure 4a), relative conduction time variations remained minimal and symmetrically distributed around zero, with no significant deviations from zero upon pulsed light delivery (cyan shaded area in Figure 4), confirming that light alone did not alter conduction properties in the absence of Ziapin2.

In contrast, upon treatment with Ziapin2 (Figure 4b), pulsed light elicited a marked and transient increase in conduction times over the interelectrode intervals in which the action potential wavefront was situated when the light was on. In the example shown in Figure 4, the relative conduction time variation during the illumination window was 0.1 ± 0.01, higher than compared to the same strand without Ziapin2 (−0.001 ± 0.003). This effect was significant (as the mean ± 2 S.E.M. interval clearly did not include zero) and tightly time-locked to the light pulses, depending on the relative timing of stimulation with respect to ongoing activation. Similar results were observed in all the analyzed strands (*n* = 5). Notably, light stimulation delivered as the activation wave reached a given region caused transient conduction delays, highlighting the sensitivity of the system to the precise timing of optical stimulation. These findings confirm that short, pulsed light stimuli can further dynamically modulate conduction in the presence of Ziapin2.

## 3. Discussion

### 3.1. Ziapin2 Reduces Conduction Velocity in the Dark

In the dark, when the compound resides in its trans conformation, we observe a clear reduction in CV, consistent with a capacitance-driven effect. An increase in C_m_ prolongs the membrane time constant (τ), which slows the upstroke of the action potential and the activation of downstream cells, thus reducing CV [1]. These observations suggest that passive capacitive membrane modulation is a primary contributor to conduction slowing, although additional effects cannot be excluded.

In cardiomyocytes, Ziapin2-induced changes in membrane thickness also modulate stretch-activated channels [30]. However, while we cannot entirely rule out a mechanosensitive modulation of sodium channels, this appears unlikely. Mechanosensitive enhancement of I_Na_, as reported by Beyder and colleagues [32], would be expected to enhance peak I_Na_ through accelerated activation kinetics and thus increase, rather than decrease, CV. Moreover, this effect requires substantial membrane deformation, far exceeding the local structural changes induced by our compound.

### 3.2. The Paradoxical Effect of Light on Ziapin2-Modulated Conduction

Unexpectedly, illumination further slowed conduction, even though the predicted decrease in C_m_ following Ziapin2 photoisomerization to the cis state would theoretically favor faster conduction [26]. This paradox suggests that light effects are not solely mediated by C_m_ changes but may involve transient perturbations of the transmembrane potential (V_m_) occurring at the light onset. Previous experiments in isolated human induced pluripotent stem cell-derived cardiomyocytes (hiPSC-CMs) and adult mouse ventricular myocytes (AMVMs) have consistently documented a biphasic V_m_ response to sub-threshold photostimulation (the one used in our experiments see Figure S2): an initial rapid hyperpolarization of ~4–5 mV at light onset, followed by a slower depolarization (~1 mV) occurring ~250 ms after light offset [30,31].

### 3.3. Mechanistic Insights into Light-Induced Reduction of Conduction Velocity

The mechanisms underlying the light-induced slowing of CV are complex and not yet fully understood, but several potential explanations can be proposed: The initial hyperpolarization following Ziapin2 photoisomerization may transiently shift V_m_ away from threshold, lowering excitability and delaying downstream activation. This brief hypoexcitable state may suffice to impair wavefront propagation. The subsequent small and delayed depolarization is unlikely to restore full excitability and may even promote Na^+^ channel inactivation in partially recovered or heterogeneous regions. Furthermore, for short light pulses, the slow depolarizing phase occurring after light offset should, in principle, have no impact on the propagating wavefront. In our experiments, the conduction times remained increased for about 10 ms after the light had been turned off, albeit to a lesser extent than during illumination (see Figure 4b, data corresponding to the intervals coded in purple, brown and grey). This brief persistence could reflect a transient electrotonic mismatch across the network before full CV recovery.

Additionally, despite the fact that during light stimulation, Ziapin2 is held predominantly in the cis conformation, thermal relaxation may revert a fraction of molecules to the trans conformation. This creates a steady state, in which the trans and cis configurations are in dynamic equilibrium under light irradiation. Such ongoing isomerization could lead to persistent or oscillatory changes in C_m_ (and V_m_), producing a sustained conduction slowing that outlasts the initial hyperpolarizing response. These effects collectively alter the balance between the depolarizing current generated by active cells and the electrotonic load of the downstream tissue, the so-called source–sink relationship, which in turn affects CV [1]. Moreover, adjusting the intensity of the light to be perfectly homogeneous is challenging. Thus, the light-induced slowing of CV possibly also reflects a transient, spatially heterogeneous disturbance in excitability and current dynamics, highlighting the complex bioelectrical impact of Ziapin2 photoisomerization. Finally, we cannot exclude that Ziapin2 might also modulate gap junctional coupling, which would also affect CV.

### 3.4. Ziapin2: Potential Applications in Cardiac Conduction Modulation

The ability to modulate cardiac conduction via passive membrane mechanisms opens several compelling applications: (i) by selectively modulating membrane capacitance and excitability, Ziapin2 can experimentally induce slow conduction or block. This allows researchers to isolate and study the arrhythmogenic role of impaired conduction, separate from any effects on ion channels, particularly in the context of reentry and fibrillation; (ii) the compound offers a powerful tool to dissect how passive membrane properties govern excitability and propagation, particularly when combined with pharmacological or genetic perturbations; (iii) Ziapin2 is well suited for use in advanced cellular models, including hiPSC-CM monolayers, engineered cardiac tissues, and organ-on-chip platforms [33,34]. Its compatibility with optical stimulation and recording techniques enables non-invasive control and high-resolution mapping of bioelectrical activity across multicellular preparations; (iv) considering the clinical needs in arrhythmia management, including atrial and ventricular arrhythmias, Ziapin2 may offer a strategy for precise, localized control of cardiac conduction, potentially preventing or interrupting reentrant circuits through targeted illumination. The practical translation of this approach will require addressing challenges such as developing safe and effective methods for local myocardial delivery, which have been recently discussed in detail by our group [20].

### 3.5. Ziapin2 in Cardiac Conduction Modulation: Pros & Cons

Unlike optogenetic approaches, which rely on genetically encoded light-sensitive proteins to directly control ion channels and cellular activity [35], and classical photopharmacological strategies, which modulate G protein-coupled receptors, ion channels, or signaling pathways to achieve light-dependent control of cardiac function [36,37,38], Ziapin2 modulates conduction velocity in a non-genetic manner by primarily altering cellular passive membrane properties [26]. Nevertheless, its main limitation remains intrinsic light-independent activity, which reduces conduction velocity even in the absence of illumination. Such baseline effects could pose safety concerns in arrhythmia-prone tissues, where conduction slowing may exacerbate electrical instability. However, it is important to consider that the magnitude of these effects is likely concentration- and cell type–dependent, indicating that they may vary across experimental settings and tissue models. This observation underscores the need for careful dose–response characterization of Ziapin2, as well as systematic evaluation of the optimal concentration and potential toxicity thresholds across different cellular contexts.

In parallel, the development of next-generation derivatives with minimal dark activity and enhanced light sensitivity will be crucial to fully exploit this strategy for future applications [20]. One promising strategy is to shift from a mechanism based predominantly on a mechano-modulation of membrane thickness to one relying on photoinduced changes in dipole moment. By engineering derivatives whose trans configuration exhibits minimal interaction with C_m_, while the cis configuration induces a strong dipolar perturbation upon light activation, it should be possible to suppress the undesired constitutive activity in the dark. In this regard, push–pull azobenzenes represent a compelling design direction, as they reduce the membrane-binding or dimerizing tendency of the trans isomer while maximizing the photoinduced dipolar modulation that underlies the desired optical control of C_m_ [39].

## 4. Materials and Methods

### 4.1. Patterned Cardiomyocyte Cultures on Microelectrode Arrays

Neonatal (0–24 h post-partum) or fetal (embryonic day 19.5) ventricular myocytes were isolated from the hearts of wild-type C57BL/6J mice and cultured following previously established protocols [27,28,29]. Myofibroblast proliferation was inhibited by bromodeoxyuridine [27,28,29]. The cells were cultured on microelectrode arrays (MEAs; Sensors, Actuators and Microsystems Laboratory, University of Neuchâtel, Switzerland) housed within custom-fabricated recording chambers (volume: 1 mL). All procedures involving animals were conducted in accordance with the ethical guidelines of the Swiss Academy of Medical Sciences. The MEAs consisted of 4 series of 12 aligned extracellular electrodes (diameter of 40 µm; interelectrode spacing of 0.5 mm along each series, and 1.5 mm between the series) with two stimulation dipoles at the end of each series (see Figure 1). The recording electrodes and leads were photolithographically patterned using transparent indium-tin oxide on borosilicate glass substrates, based on custom-designed layouts. Stimulation electrodes were further coated with platinum to enhance the electrode-electrolyte interface. The leads were encapsulated using a layer of silicon nitride. Cardiomyocytes were seeded at a density of 400,000/cm^2^ and their growth was patterned to form strands 100–150 µm in width and 7 mm in length aligned over the corresponding series of 12 recording electrodes. To ensure a uniform extracellular environment across preparations during electrophysiological recordings (conducted in cultures 2–3 days after seeding), the culture medium was replaced with Hanks’ Balanced Salt Solution (HBSS) immediately before measurements. Chambers were connected with a custom-built amplifier array [40] (gain: 1000×) and placed back in a temperature-controlled incubator (36 °C and 0.9% CO_2_). Recordings were started after a 60 min temperature equilibration period after introducing the MEA with the amplifier array into the incubator, and also after the application and washout of Ziapin2.

### 4.2. Pacing and Recording

The preparations were stimulated from the platinum-coated dipoles using biphasic voltage pulses of opposite polarity (amplitude: 0.5–2.5 V; duration of each phase: 1–5 ms, approximately 50% above threshold). Each preparation was initially paced for 1 min at a basic cycle length (BCL) of 300 ms. Only preparations exhibiting continuous 1:1 stimulus capture throughout the entire pacing protocol and showing stable conduction velocity (CV) after the initial minute of stimulation were included in the analysis.

Extracellular unipolar electrograms were recorded from the sets of 12 indium-tin oxide microelectrodes and sampled at 10 kHz. Activation times were defined as the time point corresponding to the minimum of the first derivative of the extracellular electrograms. CV along the strands was determined by linear regression of activation times vs. position. Only preparations displaying uniform conduction (correlation coefficient r > 0.999) were considered for further analysis.

### 4.3. Ziapin2 Synthesis and Administration

Ziapin2 was synthesized according to a previously published procedure [26]. The wild-type murine cardiomyocyte cultures were treated following an established protocol for compound uptake [30,41]. Specifically, the cells were incubated with 25 μM Ziapin2 (vehicle: DMSO, final concentration 0.6%) for 7 min at 36 °C and 0.9% CO_2_ under dark conditions to prevent premature photoactivation. After incubation, excess compound was removed through a washing step. Both the Ziapin2 delivery and wash solutions consisted of Hanks’ Balanced Salt Solution (HBSS, Gibco, Waltham, MA, USA), prewarmed to 37 °C.

### 4.4. Light Stimulation

Optical stimulation was performed using a focused LED light source (λ: 470 nm; Thorlabs, Newton, NJ, USA) positioned below the MEA. The illumination area was a circular spot of 1 cm diameter (78.5 mm^2^) with an average irradiance of 12 mW/mm^2^. Both electrical and optical stimulation (timings, durations) were configured and controlled (precision: 0.1 ms) via custom hardware and software.

### 4.5. Statistics

Normality of distribution was assessed using D’Agostino–Pearson’s normality test. To compare two sample means, either the Student’s *t*-test and the Mann–Whitney U-test were used for continuous or categorical data, respectively. A *p*-value < 0.05 was considered statistically significant; however, the actual *p*-value for the comparison was reported as an index of robustness. *n* represents the number of distinct preparations used.

## 5. Conclusions

In this study, we demonstrate that Ziapin2 can modulate cardiac conduction in neonatal or fetal murine cardiomyocyte strands, with effects suggesting contributions from both passive membrane dynamics and transient V_m_ perturbations. While these mechanistic interpretations are supported by previous literature, they remain speculative, as direct experimental verification and quantitative dissection of the underlying mechanisms are still required. Future studies should combine patch-clamp recordings, measurements of intercellular coupling, and mathematical modeling to disentangle the relative contributions of membrane capacitance modulation and V_m_ perturbations. Clarifying these aspects will be essential to fully understand Ziapin2’s mechanism of action and to harness its potential as a tool for precise, non-genetic modulation of cardiac conduction.

## Data Availability

The datasets used and/or analyzed during the current study are available from the corresponding author upon reasonable request.

## Supplementary Materials

The following supporting information can be downloaded at https://www.mdpi.com/article/10.3390/ijms262411766/s1.

## Abbreviations

The following abbreviations are used in this manuscript:

AMVMs	Adult mouse ventricular myocytes
BCL	Basic cycle length
C_m_	Membrane capacitance
CV	Conduction velocity
HBSS	Hanks’ Balanced Salt Solution
hiPSC-CMs	Human induced pluripotent stem cell-derived cardiomyocytes
I_Na_	Sodium current
MEAs	Microelectrode array
V_m_	Membrane potential
τ	Membrane time constant
