# Supplementary material for: Capacitance-Driven Modulation of Cardiac Impulse Conduction by an Intramembrane Molecular Photoswitch
Source: Int J Mol Sci. 2025 Dec 5;26(24):11766. doi: 10.3390/ijms262411766 (PMC12732376; doi:10.3390/ijms262411766)
Supplement: Supplementary file 1 [file ijms-26-11766-s001.zip › ijms-4014219-supplementary.pdf]

SUPPLEMENTARY FIGURES

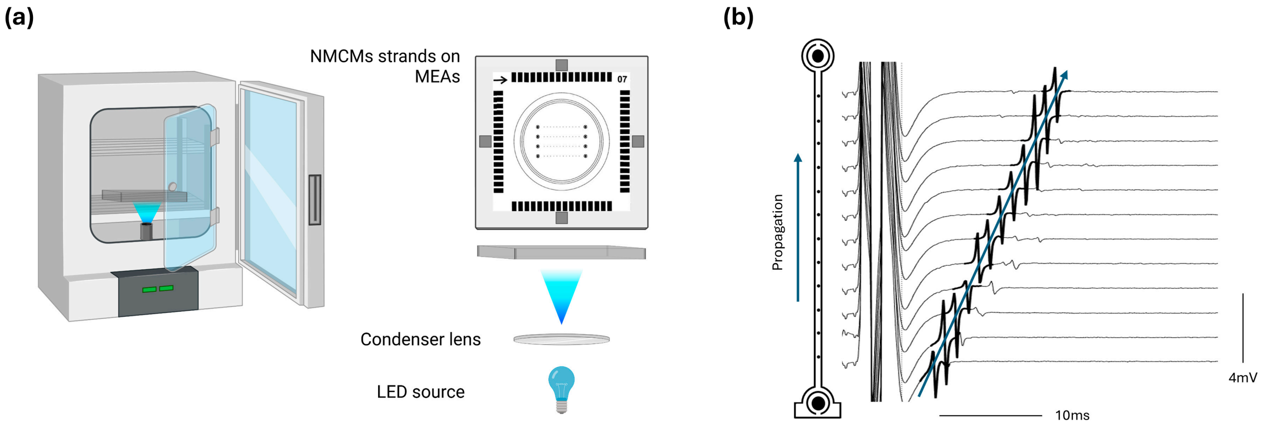

Figure S1. Experimental setup for optical stimulation of cardiac strands on MEAs. (a) Schematic representation of the experimental setup used for optical stimulation inside a cell culture incubator. Cardiac strands cultured on transparent microelectrode arrays (MEAs) were illuminated from below using a high-power 470 nm LED mounted inside the incubator. The LED produced upward illumination through the transparent MEA substrate, covering an area of approximately 78.5 mm^2 on the MEA surface. This configuration allowed relatively homogeneous light delivery across the sample, with a maximum achievable average power density of approximately 12 mW/mm^2, while maintaining controlled physiological conditions (36 °C, 0.9% CO_2). Panel (b) represents an electrogram depicting signal propagation recorded within a cardiac strand. Panel (a) was created in BioRender. Florindi, C. (2025) https://BioRender.com/qjgrpgx.

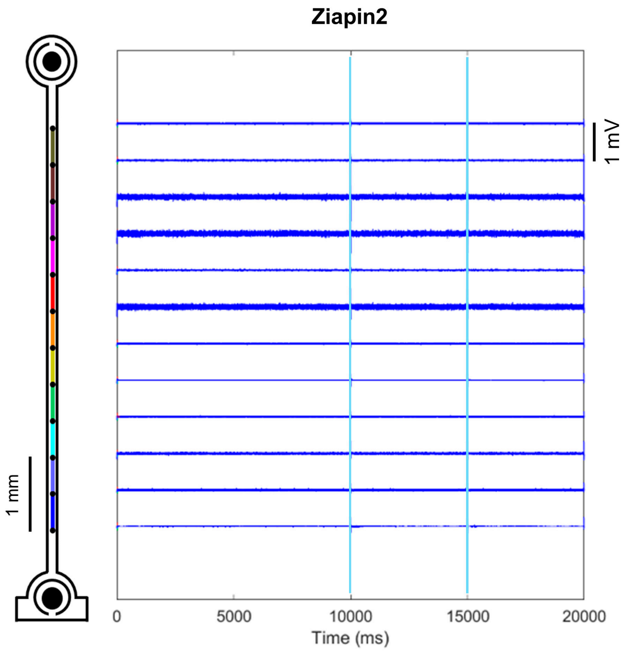

Figure S2. Optical stimulation does not elicit extracellular field potentials in the presence of Ziapin2. Representative electrogram recorded from a Ziapin2-loaded cardiac strand cultured on a MEA. Optical pulses (20 ms, 470 nm) were delivered at 10 s and 15 s, as indicated by the cyan shaded areas. Traces are vertically aligned according to electrode position along the series of electrodes (schematic on the left). Note that exposition to light caused a slow drift of the signal; this drift was removed in this figure using a high-pass filter.
